# Impact of updated regulatory guidelines on study results in contemporary uncomplicated urinary tract infection clinical trials and implications for trial conduct and drug development: a comparative analysis with EAGLE-2 and EAGLE-3

**DOI:** 10.1016/j.conctc.2025.101572

**Published:** 2025-11-18

**Authors:** Florian Wagenlehner, Keith S. Kaye, David A. Talan, Amanda J. Sheets, Nicole E. Scangarella-Oman, Emily Jarvis, Jeremy Dennison, Salim Janmohamed, Matthew Helgeson, Caroline Perry

**Affiliations:** a Justus Liebig University, Clinic for Urology, Pediatric Urology and Andrology, Rudolf Buchcheim Strasse 7, 35392, Giessen, Germany; b Rutgers Robert Wood Johnson Medical School, 125 Paterson Street, 08901, New Brunswick, NJ, USA; c David Geffen School of Medicine at UCLA, Department of Emergency Medicine, 1100 Glendon Avenue, Suite 1200–177746, Los Angeles, CA, USA; d GSK, Clinical Sciences (Infectious Diseases), 120 South Collegeville Road, Collegeville, PA, 19426-0989, USA; e GSK, Clinical Microbiology (Infectious Diseases Research), 120 South Collegeville Road, Collegeville, PA, 19426-0989, USA; f GSK R&D, Biostatistics, Gunnels Wood Road, Stevenage, SG1 2NY, UK; g GSK HQ, 79 New Oxford Street, London, WC1A 1DG, UK; h GSK R&D, Clinical Research and Early Programs, Respiratory Immunology, and Inflammation Research Unit, 79 New Oxford Street, London, WC1A 1DG, UK; i GSK, Global Medical Affairs, Antibiotics, 120 South Collegeville Road, Collegeville, PA, 19425-0989, USA; j GSK, Clinical Research Infectious Diseases, 1250 South Collegeville Road, Collegeville, PA, 19425, USA

**Keywords:** Clinical success, Composite primary endpoint, Investigator-assessed clinical response, Resolution or near resolution, Therapeutic failure, Uncomplicated urinary tract infections

## Abstract

**Aim:**

To understand the impact of current regulatory guidance for non-inferiority, randomized controlled trials (RCTs) in uncomplicated urinary tract infection (uUTI) on efficacy outcomes.

**Methods:**

EAGLE-2 and EAGLE-3 were phase 3, non-inferiority RCTs of oral gepotidacin (1500 mg twice daily for 5 days) vs nitrofurantoin (100 mg BID for 5 days) in females with uUTI. The composite (clinical and microbiological) primary endpoint, therapeutic response (success or failure), was assessed at test-of-cure (day 10–13) in participants with nitrofurantoin-susceptible uropathogens (≥10^5^ colony forming units/mL). Success required symptom resolution plus microbiological eradication; missing data or additional antibacterial use were considered failure. EAGLE-2/-3 results were compared with historic nitrofurantoin RCTs and exploratory endpoints – symptom “resolution or near resolution” (one mild symptom remaining) and investigator-assessed clinical response (IACR) – were used as alternative measures of clinical success.

**Results:**

Nitrofurantoin therapeutic success was substantially lower in EAGLE-2/-3 (47 %/44 %) than historic studies (61–94 %) using different endpoints. Clinical success rates based on “resolution or near resolution” of symptoms were: 78.3 %/77.2 % (EAGLE-2) and 75.7 %/75.3 % (EAGLE-3) for gepotidacin/nitrofurantoin, respectively. IACR rates were: 84.5 %/82.6 % (EAGLE-2) and 75.5 %/76.4 % (EAGLE-3) (post hoc analysis).

**Conclusion:**

Differences in primary endpoint success criteria need to be considered when comparing contemporary and historic uUTI RCTs.

The trials are registered at Clinicaltrials.gov (EAGLE-2, NCT04020341; EAGLE-3, NCT04187144).

## Highlights

• Rates of nitrofurantoin clinical success differed in EAGLE-2/-3 vs historic studies.
• Composite primary endpoints in uUTI trials contribute to lower success rates.
• The method of determining clinical success impacts outcome assessment.
• There is a need for validated measures of clinical success in uUTI clinical trials.

## 1 Introduction

Historically, randomized controlled trials (RCTs) of antibacterials for the treatment of uncomplicated urinary tract infections (uUTIs) have used a variety of primary endpoints, with varying definitions of treatment success, methodologies, and timings of assessment [[1], [2], [3], [4]]. Current guidance for RCTs in uUTI from the US Food and Drug Administration (FDA) [5] and European Medicines Agency (EMA) [6] recommends a composite primary endpoint of clinical and microbiological response, assessed at a fixed timepoint after randomization, with success defined as full symptom resolution (clinical success) plus reduction of the baseline qualifying uropathogen(s) from ≥10^5^ to <10^3^ colony forming unit [CFU]/mL on urine culture (microbiological success) without additional systemic antimicrobial use [[5], [6], [7]]. This definition of success has not been fully validated against long-term sustained clinical cure. Moreover, the subjective nature of clinical response assessments can lead to variation in clinical response rates across sites and studies.

Gepotidacin is a novel, first-in-class, triazaacenaphthylene, bactericidal antibacterial that inhibits bacterial DNA replication by a distinct binding site, unique mechanism of action, and well-balanced inhibition (for most uUTI uropathogens) of two enzymes (DNA gyrase and topoisomerase IV), where a gepotidacin target-specific mutation in a single enzyme does not significantly impact susceptibility [[8], [9], [10]]. These characteristics provide in vitro activity against most strains of uropathogens, including isolates resistant to current antibacterials [11].

Gepotidacin was found to be non-inferior to current first-line uUTI treatment nitrofurantoin in EAGLE-2 (NCT04020341) and EAGLE-3 (NCT04187144) – two phase 3 RCTs each with a composite primary endpoint of therapeutic response at test-of-cure (TOC) in line with current FDA and EMA guidance [12]. Based on these results, gepotidacin was recently approved by the FDA and UK Medicines and Healthcare products Regulatory Agency for the treatment of female adults and paediatric patients 12 years of age and older weighing at least 40 kg with uUTI [[13], [14], [15]].

The success rates of nitrofurantoin in EAGLE-2 and EAGLE-3 were much lower than historic RCTs, which may be due to the stringency of the updated endpoint recommendations. Per the current guidelines for non-inferiority RCTs in uUTI, combined response should be evaluated in participants with all qualifying uropathogens (≥10^5^ CFU/mL) demonstrating in vitro susceptibility to the comparator drug at baseline, a condition not necessarily reflective of empirical treatment in practice, where urine cultures are not routinely performed [16]. Additionally, patients should not be excluded from the primary analysis population based on events that occur after randomization (e.g. loss to follow-up, following the intent-to-treat [ITT] principle to minimize potential bias in the treatment effect estimate by removing patients based on events that occur after randomization) [5]. Therefore, in registrational trials, subjects with missing endpoint data are typically treated as failures, with sensitivity or worst-/best-case analyses conducted to assess the impact of handling of missing data to the primary efficacy results [1,17].

The impact of the updated endpoint guidance was recently demonstrated by Grant et al., 2023 who reanalyzed data from a historic trial of nitrofurantoin and fosfomycin using the most recent FDA guidance and found a marked reduction in success rates with the updated endpoint vs the original primary endpoint of the trial (clinical response at day 28 ± 7 post-treatment completion, with clinical success defined as the resolution of symptoms without the need for additional antibacterial use) [1,17]. In their reanalysis, 59 % (61/103) and 57 % (62/108) of women treated with nitrofurantoin and fosfomycin, respectively, achieved therapeutic success at Day 14, compared with 70 % (171/244) and 58 % (139/241) of patients in each group who met treatment success criteria in the original analysis [1,17]. Reanalysis necessitated the removal of participants who no longer qualified for the analysis and included participants with missing data as therapeutic failures, when previously study participants with missing data were excluded from analysis [17].

In EAGLE-2 and EAGLE-3, the inherent nature of the composite endpoint, the stringency of individual clinical and microbiological success criteria, and the impact of missing central microbiology laboratory data and out-of-window assessments contributed to discordant responses (success combined with concurrent failure in either of the individual clinical and microbiological response endpoints, resulting in overall therapeutic failure); this, in turn, impacted the observed combined success rates [12]. Here, we compare the EAGLE-2 and EAGLE-3 primary efficacy outcomes with older RCTs assessing nitrofurantoin for uUTI, and explored the impact of applying clinical response criteria similar to those used in historical studies on the EAGLE-2/-3 clinical and composite (therapeutic) success rates.

## 2 Materials and methods

### 2.1 Study overview

EAGLE-2 and EAGLE-3 were global, phase 3, double-blind non-inferiority (10 % margin) RCTs of oral gepotidacin (1500 mg twice daily for 5 days) vs nitrofurantoin (100 mg twice daily for 5 days) in females (aged ≥12 years) with uUTI [12]. Patients were eligible to participate if they had ≥2 symptoms of uUTI and had evidence of urinary nitrite and/or pyuria (>15 white blood cells/high-power field or 3+/large leukocyte esterase). Randomized participants who received ≥1 dose of study drug and had qualifying uropathogens (≥10^5^ CFU/mL), which all demonstrated in vitro susceptibility to nitrofurantoin (NTF-S), were included in the microbiological ITT (micro-ITT) NTF-S population.

Adhering to regulatory guidance, the primary endpoint of both trials was therapeutic response at the TOC visit (day 10–13), with success defined as full symptom resolution (total symptom score = 0) plus microbiological eradication (from ≥10^5^ to <10^3^ CFU/ml) without use of additional non-study systemic antibacterials through TOC. Patients also attended an on-therapy (day 2–4) and follow-up (day 25–31) visit.

Individual symptom severity was quantified using a four-point scale based on the impact that each symptom (dysuria, frequency, urgency, and lower abdominal/suprapubic pain) had on everyday activities: “0” absence of symptom; “1” mild, symptom easily tolerated, causing minimal discomfort, and no interference with everyday activities; “2” moderate, symptom sufficiently discomforting to interfere with normal everyday activities; or “3” severe, symptom prevents normal everyday activities [18]. A patient's total symptom score was programmatically derived from their individual symptom scores (maximum score of 12).

### 2.2 Analyses

Endpoints, analysis populations, and success rates were compared descriptively between the EAGLE-2 and EAGLE-3 trials and 10 historic RCTs of nitrofurantoin in uUTI. Nine of the 10 historic studies were identified via a previously conducted systematic review of publications up to December 10, 2016 [19]. A supplemental systematic literature review (SLR) was conducted using the same inclusion/exclusion criteria as previously described [19] to identify additional RCTs published up to August 31, 2019, corresponding with when the 2019 FDA uUTI guidance was published. Using the Medline (PubMed, US National Library of Medicine, National Institutes of Health) and Embase (Elsevier) search engines, broad literature searches were performed to identify, screen, determine eligibility and select studies (RCT or open-label studies) of nitrofurantoin treatment in females with uUTI. Publications identified in the search were excluded if one or more of the following criteria were met as previously described: not published in English language; observational studies; narrative reviews; case reports/series; animal studies; pediatric clinical trials; UTI prevention or prophylaxis studies; clinical trials of asymptomatic bacteriuria; or studies with a primary objective describing the pharmacokinetics or pharmacodynamics of nitrofurantoin (without efficacy data) [19]. The supplemental SLR identified 27 publications in PubMed and 34 publications in Embase. After duplicates were removed, at stage 1 screening, 53 of 54 unique publications were excluded based on the inclusion/exclusion criteria and one publication was taken to full text review. After full text review, one publication of a nitrofurantoin RCT in uUTI (Huttner 2018 [1]) was identified as meeting all inclusion/exclusion criteria (Supplementary Fig. 1).

To explore endpoints that are potentially more clinically meaningful, two additional methods of determining clinical success in EAGLE-2 and EAGLE-3 were assessed. Firstly, in a pre-specified analysis, clinical success was defined as a total symptom score of 0 (full resolution of symptoms) or 1 (one mild symptom existing), with no new uUTI symptoms or additional confounding systemic antibacterial use; this definition of clinical success (resolution or near resolution of symptoms) was also used to derive therapeutic success, and aligned the data as much as possible with previous studies (where clinical improvement was included in definitions of success) and clinical practice [1]. This assessment was limited to clinical and therapeutic success as no alterations were made to the definition of microbiological success. Secondly, investigator assessed clinical response (IACR) was defined as sufficient resolution of symptoms such that, in the opinion of the investigator, no additional antibacterial treatment was required. The exploratory IACR endpoint was assessed post hoc in the subset of participants in the micro-ITT NTF-S population enrolled under protocol amendment 1 (EAGLE-2) and 2 (EAGLE-3), for whom IACR data were collected. For the same subset of participants, rates of clinical success (based on total symptom scores showing resolution without additional confounding systemic antibacterial use) were calculated post hoc for a direct comparison with rates of IACR.

Treatment differences (gepotidacin–nitrofurantoin) for comparisons of therapeutic, clinical, and microbiological success rates at TOC were calculated using the Miettinen–Nurminen (score) method adjusted for age and history of uUTI recurrence in the individual studies and also for study in the pooled analysis. Participants with missing outcomes data were considered failures in the above analyses.

## 3 Results

Across the EAGLE-2 and EAGLE-3 trials and treatment groups, rates of therapeutic success were 9.2–19.6 % and 13.5–20.8 % lower than rates of clinical and microbiological success, respectively (Table 1). Lack of symptom resolution (often with microbiological eradication) accounted for therapeutic failure in 26.0 % of patients assigned to gepotidacin and in 30.7 % assigned to nitrofurantoin (Table 2). Lack of microbiological eradication (baseline uropathogen persisted or recurred after earlier eradication) accounted for 13.2 % and 27.2 % of responses in gepotidacin- and nitrofurantoin-assigned patients, respectively. Clinical and microbiological failure responses did not always correlate, with lack of symptom resolution combined with lack of microbiological eradication only reported by 7.2 % and 13.1 % of gepotidacin and nitrofurantoin-assigned patients, respectively. Overall, few patients received additional antibacterial treatment for uUTI (3.7 % and 4.9 % of gepotidacin and nitrofurantoin-assigned patients, respectively). Missing data at TOC (including out-of-window assessments) was responsible for therapeutic failure classification in 10.5 % and 7.5 % of gepotidacin and nitrofurantoin-assigned patients, respectively (Table 2).

**Table 1:** Therapeutic, clinical, and microbiological response at TOC in EAGLE-2 and EAGLE-3 (micro-ITT NTF-S population).

	EAGLE-2		EAGLE-3		Pooled data	
	Gepotidacin 1500 mg BID (*n* = 336)	Nitrofurantoin 100 mg BID (*n* = 298)	Gepotidacin 1500 mg BID (*n* = 292)	Nitrofurantoin 100 mg BID (*n* = 275)	Gepotidacin 1500 mg BID (*n* = 628)	Nitrofurantoin 100 mg BID (*n* = 573)
Therapeutic success	174 (51.8)	140 (47.0)	172 (58.9)	121 (44.0)	346 (55.1)	261 (45.5)
Treatment difference,% (95 % CI)^a^	5.3 (−2.4, 13.0)		14.4 (6.4, 22.4)		9.6 (4.1, 15.2)	
Therapeutic failure	162 (48.2)	158 (53.0)	120 (41.1)	154 (56.0)	282 (44.9)	312 (54.5)
Clinical success, microbiological failure	50 (14.9)	56 (18.8)	27 (9.2)	54 (19.6)	77 (12.3)	110 (19.2)
Clinical failure, microbiological success	70 (20.8)	59 (19.8)	41 (14.0)	37 (13.5)	111 (17.7)	96 (16.8)
Clinical failure, microbiological failure	42 (12.5)	43 (14.4)	52 (17.8)	63 (22.9)	94 (15.0)	106 (18.5)
Clinical success	224 (66.7)	196 (65.8)	199 (68.2)	175 (63.6)	423 (67.4)	371 (64.7)
Treatment difference,% (95 % CI)^a^	1.5 (−5.8, 8.8)		4.3 (−3.4, 12.0)		2.8 (−2.4, 8.1)	
Clinical failure	112 (33.3)	102 (34.2)	93 (31.8)	100 (36.4)	205 (32.6)	202 (35.3)
Clinical improvement (without resolution)	82 (24.4)	75 (25.2)	51 (17.5)	68 (24.7)	133 (21.2)	143 (25.0)
Clinical worsening (or no change from baseline)	9 (2.7)	16 (5.4)	20 (6.8)	17 (6.2)	29 (4.6)	33 (5.8)
Unable to determine^b^	21 (6.3)	11 (3.7)	22 (7.5)	15 (5.5)	43 (6.8)	26 (4.5)
Microbiological success	244 (72.6)	199 (66.8)	213 (72.9)	158 (57.5)	457 (72.8)	357 (62.3)
Treatment difference,% (95 % CI)^a^	6.0 (−1.2, 13.1)		15.5 (7.9, 23.1)		10.4 (5.2, 15.6)	
Microbiological failure	92 (27.4)	99 (33.2)	79 (27.1)	117 (42.5)	171 (27.2)	216 (37.7)
Microbiological persistence	15 (4.5)	21 (7.0)	13 (4.5)	31 (11.3)	28 (4.5)	52 (9.1)
Microbiological recurrence^c^	36 (10.7)	52 (17.4)	19 (6.5)	52 (18.9)	55 (8.8)	104 (18.2)
Unable to determine^d^	41 (12.2)	26 (8.7)	47 (16.1)	34 (12.4)	88 (14.0)	60 (10.5)

Data are n (%) unless otherwise stated.

Abbreviations: BID: twice daily; CI: confidence interval; CFU: colony forming unit; micro-ITT NTF-S: microbiological intent-to-treat nitrofurantoin-susceptible; TOC: test-of-cure; uUTI: uncomplicated urinary tract infection.

a Difference: gepotidacin – nitrofurantoin calculated using Miettinen–Nurminen (score) method adjusting for age and history of uUTI recurrence based on case report form data (for the individual studies) or adjusting for age, history of uUTI recurrence, and study (for the pooled analysis).

b A clinical outcome of “unable to determine” was defined as a missing baseline score (for patients who did not meet the clinical success definition), or a missing TOC assessment, or when a patient received other systemic antimicrobials not for uncomplicated UTI before the TOC visit (unless clinical worsening criteria were met).

c Microbiological recurrence was defined as uropathogens ≥10^3^ CFU/ml after eradication at the on-therapy visit (without receipt of other systemic antibacterial before the TOC visit).

d A microbiological outcome of “unable to determine” was defined as a missing urine culture result or when a patient received other systemic antibacterial before the TOC visit.

**Table 2:** Reasons for therapeutic failure at TOC (micro-ITT NTF-S population).

	EAGLE-2		EAGLE-3		Pooled data	
	Gepotidacin (*n* = 336)	Nitrofurantoin (*n* = 298)	Gepotidacin (*n* = 292)	Nitrofurantoin (*n* = 275)	Gepotidacin (*n* = 628)	Nitrofurantoin (*n* = 573)
Therapeutic failure at TOC	162 (48.2)	158 (53.0)	120 (41.1)	154 (56.0)	282 (44.9)	312 (54.5)
Lack of symptom resolution (improved, no change, or worsened)	92 (27.4)	91 (30.5)	71 (24.3)	85 (30.9)	163 (26.0)	176 (30.7)
With microbiological eradication	70 (20.8)	59 (19.8)	41 (14.0)	37 (13.5)	111 (17.7)	96 (16.8)
Lack of microbiological eradication (persisted/recurred)	51 (15.2)	73 (24.5)	32 (11.0)	83 (30.2)	83 (13.2)	156 (27.2)
With symptom resolution	34 (10.1)	49 (16.4)	21 (7.2)	46 (16.7)	55 (8.8)	95 (16.6)
Lack of symptom resolution and lack of microbiological eradication	19 (5.7)	31 (10.4)	26 (8.9)	44 (16.0)	45 (7.2)	75 (13.1)
Receipt of non-study antibacterial for uUTI through TOC	5 (1.5)	13 (4.4)	18 (6.2)	15 (5.5)	23 (3.7)	28 (4.9)
Reasons for unable to determine responses						
Any missing data at TOC driving therapeutic response^a^	34 (10.1)	20 (6.7)	32 (11.0)	23 (8.4)	66 (10.5)	43 (7.5)
Use of potentially confounding systemic antibacterial not for uUTI prior to non-missing TOC assessment^b^	2 (0.6)	1 (0.3)	1 (0.3)	1 (0.4)	3 (0.5)	2 (0.3)

Data are n (%).

Reasons for therapeutic failure at TOC were assessed post hoc; more than one failure reason may apply.

Abbreviations: micro-ITT NTF-S: microbiological intent-to-treat nitrofurantoin-susceptible; TOC: test-of-cure; uUTI: uncomplicated urinary tract infection.

a Missing data included out-of-window assessments.

b Participants who also received non-study systemic antibacterial for uUTI through TOC were not counted in this ‘unable to determine’ row.

Nitrofurantoin therapeutic success rates in EAGLE-2 and EAGLE-3 were substantially lower than previous studies which reported 61–94 % at similar or later timepoints (Table 3). None of the RCTs included used a composite endpoint. Other notable study design differences include how clinical response was assessed. Huttner et al., for example, defined clinical response by investigator assessment of complete symptom resolution (without symptom scoring) [1], while Gupta et al. defined clinical cure as no requirement for a new antibacterial to treat the uUTI [2]; the rates of clinical success in these studies were 70 % and 84 %, respectively. If the prior clinical cure criteria had been used in EAGLE-2/-3, substantially higher success rates would have been observed at TOC as only 1.5/6.2 % of patients receiving gepotidacin and 4.4/5.5 % receiving nitrofurantoin required additional antibacterials for their uUTI during study participation through TOC. Study eligibility and analysis population criteria also differed between historic nitrofurantoin RCTs and the most recent guidance. Some historic RCTs required just a single symptom of uUTI for inclusion and in several studies the bacteriuria threshold necessary for inclusion was <10^5^ CFU/ml (Table 3) [[1], [2], [3]]. By contrast, current guidance suggests ≥2 symptoms for study eligibility, with a true bacterial pathogen (generally a single species in pure culture) at ≥10^5^ CFU/ml for efficacy analysis population criteria [5].

**Table 3:** Comparisons between EAGLE-2/-3 and historic RCTs of nitrofurantoin in uUTI.

	Efficacy endpoint(s)^a^^,^^b^	Study eligibility	Analysis population	Nitrofurantoin efficacy results
EAGLE-2/EAGLE-3 [12], participants received 100 mg of nitrofurantoin BID for 5 days	Therapeutic response (Day 10–13) *Full symptom resolution (clinical success) and microbiological eradication (*≥*10*^*5*^ → <*10*^*3*^ *CFU/ml)*	Female, ≥12 years, ≥2 uUTI symptoms, urinary nitrite, and/or pyuria	≥1 study drug dose, ≥1 qualifying uropathogen^c^ NTF-S	Therapeutic success (micro-ITT NTF-S population):
				EAGLE-2: 47.0 % (140/298)
				EAGLE-3: 44.0 % (121/275)
				Clinical success:
				EAGLE-2: 65.8 % (196/298)
				EAGLE-3: 63.6 % (175/275)
				Microbiological success:
				EAGLE-2: 66.8 % (199/298)
				EAGLE-3: 57.5 % (158/275)
Huttner 2018 [1], participants received 100 mg of nitrofurantoin TID for 5 days	Clinical response (Day 28)	Female, adult (≥18 years), ≥1 uUTI symptom, urinary nitrite/pyuria	≥1 study drug dose, no resistance to study drug, ≥80 % regimen adherence	Clinical resolution: 70 % (171/244)
	*Complete resolution of symptoms (investigator assessed)*			
Gupta 2007 [2], participants received 100 mg of nitrofurantoin BID for 5 days	Clinical cure rate (Day 30)	Female, adult (18–45 years), ≥1 uUTI symptom, positive urine culture ≥10^2^ CFU/ml	≥1 study drug dose	Clinical cure rate: 84 % (134/160)
	*No new antibacterials required for UTI symptoms*			
Christiaens 2002 [3], participants received 100 mg of nitrofurantoin QID for 3 days	1. Symptomatic cure^d^ (Days 3 & 7) *Symptom resolution (investigator assessed)* 2. Symptomatic improvement (Days 3 & 7) *Few symptoms (investigator assessed)* 3. Bacteriologic cure (Days 3 & 7) ≥*10*^*5*^ → <*10*^*5*^ *CFU/ml*	Female, 15–54 years, presenting to primary care with UTI symptoms, pyuria	Received ≥1 dose of study drug, uropathogen ≥10^5^ CFU/ml	1. Symptomatic cure: Day 3: 37 % (13/35); Day 7: 70 %^e^ (24/34) 2. Symptomatic improvement (not cure): Day 3: 40 % (14/35); Day 7: 18 % (6/34)^f^ 3. Bacteriologic cure:Day 3: 81 % (21/26); Day 7: 74 % (17/23)
Stein 1999 [4], participants received 100 mg of nitrofurantoin OD for 7 days	Treatment efficacy was assessed by both bacteriologic and clinical response 5–11 days after the initial treatment dose (visit 2) and 5–11 days (visit 3) and 4–6 weeks (visit 4) after the last day of medication: 1. Bacteriologic cure ≥*10*^*5*^ → <*10*^*4*^ *CFU/ml* 2. Clinical responses were defined as cure (*elimination of all pretherapy symptoms),* improvement *(most but not all symptoms improved or absent),* or failure *(not improved from initial assessment).* Any patient treated with an alternative antibiotic was considered a clinical failure.	Female, ≥12 years, clinical UTI symptoms	Received ≥1 dose of study drug, uropathogen ≥10^5^ CFU/ml, ≥1 post-treatment efficacy assessment	1. Bacteriologic cure: visit 2: 86.3 % (189/219) visit 3: 80.9 % (127/157) visit 4: 91.1 % (102/112) 2. Clinical response (cure/improvement): visit 2: 84.1 % (206/245)/10.6 % (26/245) visit 3: 88.9 % (193/217)/2.8 % (6/217) visit 4: 91.7 % (165/180)/1.7 % (3/180)
Iravani 1999 [25], participants received 100 mg of nitrofurantoin BID for 7 days	Bacteriological response: eradication was defined as ≥10^3^ → <10^3^ CFU/ml, 4–10 days after completion of therapy without a superinfection at any time during therapy or up to 10 days after completion of therapy and requiring additional antibacterial therapy	Adult females with uUTI symptoms and pyuria, positive urine culture ≥10^3^ CFU/ml within 48 h prior to treatment	Participants with diagnosis established by clinical signs and symptoms; single causative pathogen ≥10^3^ CFU/ml; urine culture repeated 4–10 days after therapy; study drug taken for seven full days unless treatment was a failure; no other antimicrobial agent active against the causative organism administered concomitantly with the study drug	Bacteriological response: Day 4–10: 86 % (153/177)^h^
Hooton 1995 [26], participants received 100 mg of nitrofurantoin QID for 3 days	Cure was defined as symptom resolution and bacteriological eradication at late post-treatment (4–6 weeks after treatment) in patients who had no post-treatment significant bacteriuria before the visit. Bacteriuria was defined as ‘significant’ at a follow-up visit if there were ***≥***10^2^ CFU/ml with symptoms or if there were ***≥***10^5^ CFU/ml without symptoms on at least 2 consecutive cultures (asymptomatic bacteriuria)	Women (≥18 years) with symptoms of uUTI attending a student health center	Uropathogen ≥10^2^ CFU/ml, met enrolment criteria, returned for the late post-treatment visit, and had not been treated with an antibiotic for reasons other than for recurrent UTI	Cure rate at late post-treatment: 4–6 weeks post treatment: 61 % (22/36)
Spencer 1994 [27], participants received 100 mg of nitrofurantoin BID for 7 days	Treatment efficacy was assessed based on clinical cure (relief of diary card symptoms) and bacteriological cure (no pathogens) at visit 2 (Day 9–15), separately	Female, adult (≥18 years), ≥1 uUTI symptom with hematuria and pyuria	All patients and patients with bacteriuria (i.e., single organism ≥10^7^ CFU/L at baseline)	Clinical cure at visit 2:
				All patients: 87.2 % (143/164)
				Patients with bacteriuria: 86.1 % (87/101)
				Bacteriological cure at visit 2:
				Patients with bacteriuria: 82.3 % (79/96)
Van Pienbroek 1993 [28], participants received 50 mg of nitrofurantoin QID for 7 days	Treatment efficacy was assessed based on the patient's judgement of clinical efficacy (cured/improved) at Days 4, 9 and 42, and by bacteriological cure <10^5^ CFU/ml by dipslide at days 9 and 42. Additionally, the practitioner assessed ‘overall efficacy’ in terms of cure, improved, failure (i.e. persistence), and relapse/reinfection based on symptoms and dipslide results through Day 42	Female, ≥18 years, symptoms of acute dysuria, stranguria and/or urinary frequency	Received ≥1 dose of drug	Patient's judgment (cured/improved): Day 4: 95 % (108/114)
				Day 9: 94 % (103/109)
				Day 42: 80 % (75/94)
				Bacteriological cure:
				Day 9: 90 %^g^
				Day 42: 87 %^g^
				Overall efficacy (cure):
				Day 42: 82 % (81/99)
Lightstone 1988 [29], participants received 100 mg of nitrofurantoin QID for 7 days	Bacteriological clearance (negative culture) 1 day after end-of-medication (Day 8) and 14 days later (Day 22)^i^	Females, aged between 18 and 70 years, symptoms of acute cystitis	Positive urine culture ≥10^5^ CFU/ml; positive urine culture sensitive to study medication.	Bacteriological clearance (*all patients with positive initial culture*):
				Day 8: 84.6 % (22/26)
				Day 22: 90.9 % (20/22)
				Bacteriological clearance (*all patients with positive initial culture sensitive to study medication*):
				Day 8: 87.5 % (14/16)
				Day 22: 93.3 % (15/16)
Meyer 1987 [30], participants received 50 mg of nitrofurantoin QID for 10 days	Subjective response (symptom relief) on Day 5 and objective microbiological response (negative culture) on Day 10^j^	Female, >16 years, symptoms of lower uUTI, Gram smear results consistent with UTI	Patients who received dose of study drug and completed the study (majority of patients had a uropathogen >10^5^ CFU/ml identified)	Symptomatic relief:
				Day 5: 100 % (26/26)
				Microbiological Response:
				Day 10: 80.8 % (21/26)^k^

Abbreviations: BID, twice daily; CFU: colony-forming units; ITT, intent-to-treat; micro-ITT, microbiological intent-to-treat; NTF-S: nitrofurantoin susceptible; OD, once daily; RCT: randomized controlled trial; spp: species; TID, three times daily; UTI: urinary tract infection; uUTI: uncomplicated urinary tract infection; QID, four times daily.

a Historical studies generally excluded patients with missing outcome data, although this was not always clearly described. EAGLE-2/-3 treated missing outcome data as failure.

b Multiple responder endpoints are presented for studies that did not specify a primary efficacy endpoint. Definitions of clinical resolution/cure varied between studies, and no quantitative symptom measures were used in the historic studies.

c Qualifying uropathogens (≥10^5^ CFU/mL): gram-negative bacilli (e.g. *Escherichia coli*, *Klebsiella pneumoniae*, and *Proteus mirabilis*) plus *Staphylococcus saprophyticus* and *Enterococcus* spp. (the latter only in pure culture).

d “Symptomatic cure” and “Symptomatic improvement” were mutually exclusive categories.

e Value correct per publication but mathematically should read 71 %.

f Patients who had symptomatic improvement at Day 3 were likely to meet the definition of “Symptomatic cure” by Day 7 (and so counted within “Symptomatic cure Day 7” category, above row) and would thus not be counted in the “Symptomatic improvement (not cure), Day 7” category.

g Per Table 1 in the publication [25], 177 is correct, though the text stated that 179 patients were evaluable for efficacy

h Counts were not reported (n/N).

i Efficacy was also assessed based on time to disappearance of symptoms (frequency and dysuria).

j Time to symptom relief was also assessed.

k The response rate was derived based on 5 of 26 patients reported as microbiological failure (2 cases of relapse and 3 cases of superinfection).

### 3.1 Exploratory analyses

**Fig. 1:**
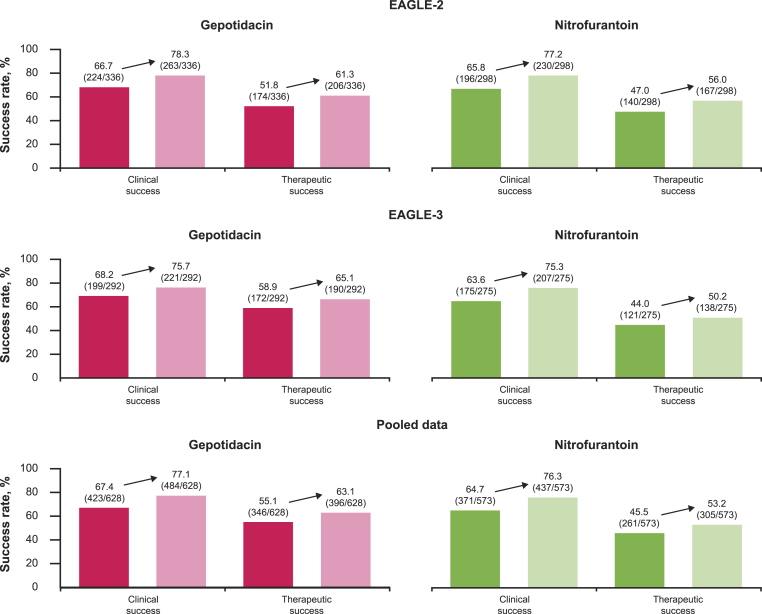
Changing the clinical success component of the EAGLE-2/-3 primary endpoint to also include “near resolution” (total symptom score ≤1) at TOC (micro-ITT NTF-S population^a^). ^a^Success was defined as ‘Resolution’ (dark pink/dark green) or ‘Resolution or near resolution’ (light pink/light green) with no new uUTI symptoms or additional confounding systemic antibacterial use through TOC. Indeterminate/missing outcomes were considered failure. Microbiological success criteria, as a component of therapeutic success, were as per the original analysis. Abbreviations: micro-ITT NTF-S: microbiological intent-to-treat nitrofurantoin susceptible isolates; TOC: test-of-cure; uUTI: uncomplicated urinary tract infection.

Data from EAGLE-2 and EAGLE-3 including “near resolution” in the definition of clinical success (micro-ITT NTF-S population) have been published previously [12]. In this analysis, the alternative measures of clinical success, resulted in increased clinical and therapeutic success. In EAGLE-2 rates increased from 66.7 % to 78.3 % for gepotidacin and from 65.8 % to 77.2 % for nitrofurantoin; and therapeutic success rates increased from 51.8 % to 61.3 % for gepotidacin and from 47.0 % to 56.0 % for nitrofurantoin (Fig. 1). In EAGLE-3, clinical success rates increased from 68.2 % to 75.7 % for gepotidacin and from 63.6 % to 75.3 % for nitrofurantoin; and therapeutic success rates increased from 58.9 % to 65.1 % for gepotidacin and from 44.0 % to 50.2 % for nitrofurantoin (Fig. 1). The missing data rates were still a contributing factor behind the modest increase in therapeutic success. Furthermore, only the clinical component of therapeutic response was altered in these analyses and microbiological success criteria (requiring a ≥2-log reduction from 10^5^ CFU/mL at baseline, a higher bacterial load than would typically be used to diagnose a uUTI when a culture is performed) continued to influence the composite endpoint.

**Fig. 2:**
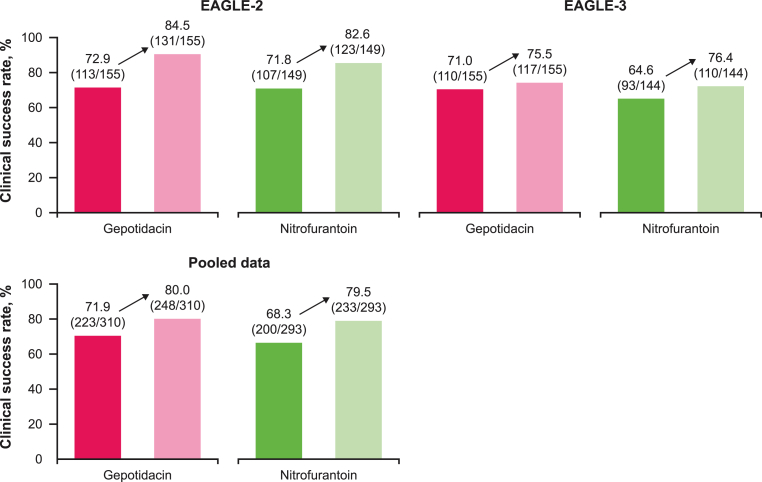
Comparison of clinical success rates with original primary endpoint definition versus Investigator-assessed clinical response at TOC (micro-ITT NTF-S subset^a^). ^a^Clinical success defined as ‘resolution’ (dark pink/dark green) and IACR (light pink/light green) was assessed post hoc in a subset of the micro-ITT NTF-S population with IACR data collected. Indeterminate/missing outcomes were considered failure. Abbreviations: IACR: investigator-assessed clinical response; ITT: intent-to-treat; micro-ITT NTF-S: microbiological intent-to-treat nitrofurantoin susceptible isolates; TOC: test-of-cure.

IACR rates at TOC in EAGLE-2/-3 were 84.5/75.5 % with gepotidacin and 82.6/76.4 % with nitrofurantoin (Fig. 2). Rates of clinical failure, as assessed by the investigator in EAGLE-2/EAGLE-3 were 5.8/14.8 % with gepotidacin and 11.4/14.6 % with nitrofurantoin; the remaining participants had indeterminate/missing responses (9.7/9.7 % with gepotidacin, 6.0/9.0 % with nitrofurantoin). In the same set of participants, rates of clinical success (based on total symptom scores of zero without confounding systemic antibacterial use) in EAGLE-2/-3 were 72.9/71.0 % for gepotidacin and 71.8/64.6 % for nitrofurantoin (post hoc analysis).

## 4 Discussion

Current RCT guidelines set a high threshold of complete symptom resolution combined with microbiological eradication for defining therapeutic success in uUTI trials. Accordingly, substantially lower success rates were observed for nitrofurantoin in EAGLE-2 and EAGLE-3 (which followed current guidelines) than those reported in prior RCTs; historically, less stringent endpoint criteria were used, with success rates of 61–94 % reported for nitrofurantoin at similar or later timepoints [1,3,4]. The current guidelines limit the ability to compare efficacy of an antibacterial treatment between historic and contemporary trials [17]. New antibacterials, such as gepotidacin, may appear less efficacious than older drugs when assessed with the latest endpoint recommendations. This difference might have an impact on healthcare professionals’ perception of the effectiveness of new drugs and treatment decision-making.

The composite primary endpoint gives equal weight to clinical and microbiological outcomes, which poses challenges. In particular, the clinical relevance of discordant outcomes, specifically clinical success without microbiological success at TOC, does not reflect clinical practice where treatment success is not typically verified with urine culture (microbiological cure) [16]. Grant et al. highlighted the importance of including microbiological endpoints in clinical trials [17]; however, its potential importance in predicting clinical relapse has not been established for uUTI. A meta-analysis of complicated urinary tract infection trials suggested that microbiological response predicts a greater likelihood of clinical failure at later study visits [20]; future analyses are needed to explore this further with EAGLE-2/-3 data.

The FDA requirement for absolute absence of specific prescribed symptoms as an indicator of clinical success for the current uUTI episode does not address the attainability of a total symptom score of 0 among patients with pre-morbid urinary symptoms such as overactive bladder or urinary incontinence [18]. Previous studies have used symptom scores above zero to define resolution; for example, Kronenberg et al. used a clinical symptom cutoff score of 2 or less as an indicator of “symptom resolution” rather than complete absence of symptoms [21]. A question remains as to whether it is realistic to expect complete resolution of symptoms by TOC, and whether a non-zero score should be validated for future studies. Notably, EAGLE-2/-3 study investigators considered patients with non-zero clinical symptom scores to be clinical successes as evidenced by the higher IACR rates. Similarly, when clinical success criteria allowed for “resolution or near resolution” of symptoms (i.e., total score ≤1), both nitrofurantoin and gepotidacin had higher levels of clinical success than when a zero score was required. While defining clinical success as “resolution or near resolution” may be more aligned with clinical practice, it is not a validated threshold and there may be more tolerance for near resolution with specific symptoms (e.g. frequency and urgency more than dysuria or lower abdominal pain). Specific symptom response criteria could be validated against 30-day generic quality-of-life tools such as the EuroQol assessment, commonly used in UTI research [22]. However, when the validated Acute Cystitis Symptom Score Questionnaire was previously used to calculate cut-off values for clinical success by comparing with quality-of-life data, the patients’ assessment by symptom thresholds alone was not sensitive as a measure of clinical cure [23]. In another study, Gupta et al. defined success as no requirement for new antibacterials for UTI symptoms [2]. If this endpoint had been used in EAGLE-2/-3, substantially higher success rates would have been observed at TOC (only 1.5/6.2 % patients receiving gepotidacin and 4.4/5.5 % receiving nitrofurantoin required additional non-study antibacterials during study participation through TOC; Table 2). While this definition is clinically useful, it may be lacking sensitivity to detect a treatment difference in the context of a registrational trial.

A composite endpoint may provide a more rigorous measure of efficacy, but the question remains whether it is correct to assume that any remaining symptom or measurable bacteriuria indicates treatment failure. That current guidance does not reflect clinical practice also begs the question: how is a clinician to treat a patient who, historically, would have been considered a therapeutic success but by the new regulatory definitions is a therapeutic failure? For new antibacterial agents, there is also interest in measuring early symptom relief that is sustained, rather than assessing therapeutic response after completion of treatment.

In the EAGLE-2/-3 studies, 10.5 % and 7.5 % of gepotidacin and nitrofurantoin-assigned participants, respectively, were therapeutic failures due to missing data (mostly microbiological data). The common practice of handling missing data as failure in registrational trials may not be the best approach. To reduce the proportion of participants with overall failure driven by missing microbiological data alone (especially when using a central laboratory in a global trial), it may be preferable to assign a microbiological response of presumed eradication or persistence based on the patient's clinical response [1,17].

There are additional considerations associated with composite endpoints that make their use in clinical trials challenging. For example, due to lower success rates (i.e., around 50 %), composite endpoints may require larger sample sizes [17], which could prohibit some companies from conducting trials. The baseline bacterial threshold of ≥10^5^ CFU/ml is also likely to result in cost implications and logistic considerations for clinical trials, as more patients must be enrolled to ensure a sufficient number meet primary analysis population criteria [17].

Our comparison of the EAGLE-2/-3 trial results with historical nitrofurantoin studies has several limitations. Firstly, the publications identified in the literature review were contingent upon the search terms and English language restrictions imposed. However, the search terms were as general as possible to identify all relevant studies, and the systematic screening of identified publications adhered to the recommendations set forth in the PRISMA statement [24]. Secondly, comparing efficacy outcomes across different studies can be problematic due to heterogeneity in study designs, including differences in patient populations, analysis populations, endpoint definitions, and statistical methodologies, all of which may influence observed response rates independently of treatment effect. Additionally, potential differences in baseline microbiology and susceptibility to nitrofurantoin across studies could influence response rates. Lastly, the inclusion of open-label studies in our SLR may have introduced bias to some of the reported efficacy outcomes.

## 5 Conclusions

Observed rates of therapeutic and clinical success with nitrofurantoin in the EAGLE-2/-3 studies, which used the stringent combined microbiological and clinical response endpoint as a threshold for uUTI treatment efficacy in line with recent regulatory guidance, were lower than in historic trials.

Exploratory analyses using clinical success criteria more in line with historic studies resulted in higher rates of clinical and therapeutic success in EAGLE-2/-3 for both gepotidacin and nitrofurantoin. Variability in assessing clinical and microbiological success should be taken into consideration when comparing results of contemporary and historic trials in uUTI, as well as interpreting the results in the context of actual patient care.

A validated methodology needs to be defined to standardize RCTs, and future prospective studies should include secondary or exploratory endpoint analyses that mirror historic studies.

## CRediT authorship contribution statement

**Florian Wagenlehner:** Writing – review & editing, Formal analysis, Data curation, Conceptualization. **Keith S. Kaye:** Writing – review & editing, Formal analysis, Conceptualization. **David A. Talan:** Writing – review & editing, Formal analysis, Conceptualization. **Amanda J. Sheets:** Writing – review & editing, Formal analysis, Conceptualization. **Nicole E. Scangarella-Oman:** Writing – review & editing, Formal analysis, Conceptualization. **Emily Jarvis:** Writing – review & editing, Formal analysis. **Jeremy Dennison:** Writing – review & editing, Formal analysis, Conceptualization. **Salim Janmohamed:** Writing – review & editing, Formal analysis, Conceptualization. **Matthew Helgeson:** Writing – review & editing, Formal analysis, Conceptualization. **Caroline Perry:** Writing – review & editing, Formal analysis, Conceptualization.

## Ethical approval

The trials were done in accordance with the Declaration of Helsinki principles and the International Council for Harmonisation guidelines for good clinical practice. The privacy rights of human subjects have been observed and informed consent was obtained for experimentation with human subjects. The protocols were approved by all necessary ethics committees and institutional review boards on April 12, 2019 (EAGLE-2) and July 31, 2019 (EAGLE-3).

## Funding

EAGLE-2 was funded in part by 10.13039/501100002066GSK and in part with Federal funds from the 10.13039/501100016206US Department of Health and Human Services; Administration for Strategic Preparedness and Response; 10.13039/100012399Biomedical Advanced Research and Development Authority (HHSO100201300011C). EAGLE-3 was funded by 10.13039/501100002066GSK.

## Declaration of competing interest

The authors declare the following financial interests/personal relationships which may be considered as potential competing interests: **Florian Wagenlehner** reports a relationship with GSK that includes: consulting or advisory and non-financial support. **Florian Wagenlehner** reports a relationship with DFG (German Research Foundation) funded research group BARICADE (FOR5427/1–466687329) that includes: speaking and lecture fees. **Florian Wagenlehner** reports a relationship with DZIF (German Center for Infection Research. Site: Giessen - Marburg - Langen) that includes: board membership. **Keith S. Kaye** reports a relationship with GSK that includes: consulting or advisory. **Amanda J. Sheets** reports a relationship with GSK that includes: employment and equity or stocks. **Nicole E. Scangarella-Oman** reports a relationship with GSK that includes: employment and equity or stocks. **Emily Jarvis** reports a relationship with GSK that includes: employment and equity or stocks. **Jeremy Dennison** reports a relationship with GSK that includes: employment and equity or stocks. **Salim Janmohamed** reports a relationship with GSK that includes: employment and equity or stocks. **Matthew Helgeson** reports a relationship with GSK that includes: employment and equity or stocks. **Caroline Perry** reports a relationship with GSK that includes: employment and equity or stocks.

If there are other authors, they declare that they have no known competing financial interests or personal relationships that could have appeared to influence the work reported in this paper.

## Data Availability

Anonymized individual participant data and study documents (including the protocols and statistical analysis plans) can be requested for further research from https://www.gsk-studyregister.com/en/.

## Glossary

BID –: twice daily
CFU –: colony forming unit
CI –: confidence interval
EMA –: European Medicines Agency
FDA –: Food and Drug Administration
IACR –: investigator-assessed clinical response
ITT –: intent-to-treat
Micro-ITT –: microbiological intent-to-treat
Micro-ITT NTF-S –: microbiological intent-to-treat nitrofurantoin-susceptible
NTF-S –: nitrofurantoin susceptible
RCT –: randomized controlled trial
TOC –: test-of-cure
UTI –: urinary tract infection
uUTI –: uncomplicated urinary tract infection
