# Supplementary material for: Impact of updated regulatory guidelines on study results in contemporary uncomplicated urinary tract infection clinical trials and implications for trial conduct and drug development: a comparative analysis with EAGLE-2 and EAGLE-3
Source: Contemp Clin Trials Commun. 2025 Nov 18;48:101572. doi: 10.1016/j.conctc.2025.101572 (PMC12701966; doi:10.1016/j.conctc.2025.101572)
Supplement: Multimedia component 1 [file mmc1.docx]

# Supplementary materials

Supplementary Figure 1. Supplemental SLR of historic RCTs of nitrofurantoin in uUTI: publication identification, screening, and inclusion of eligible publications.


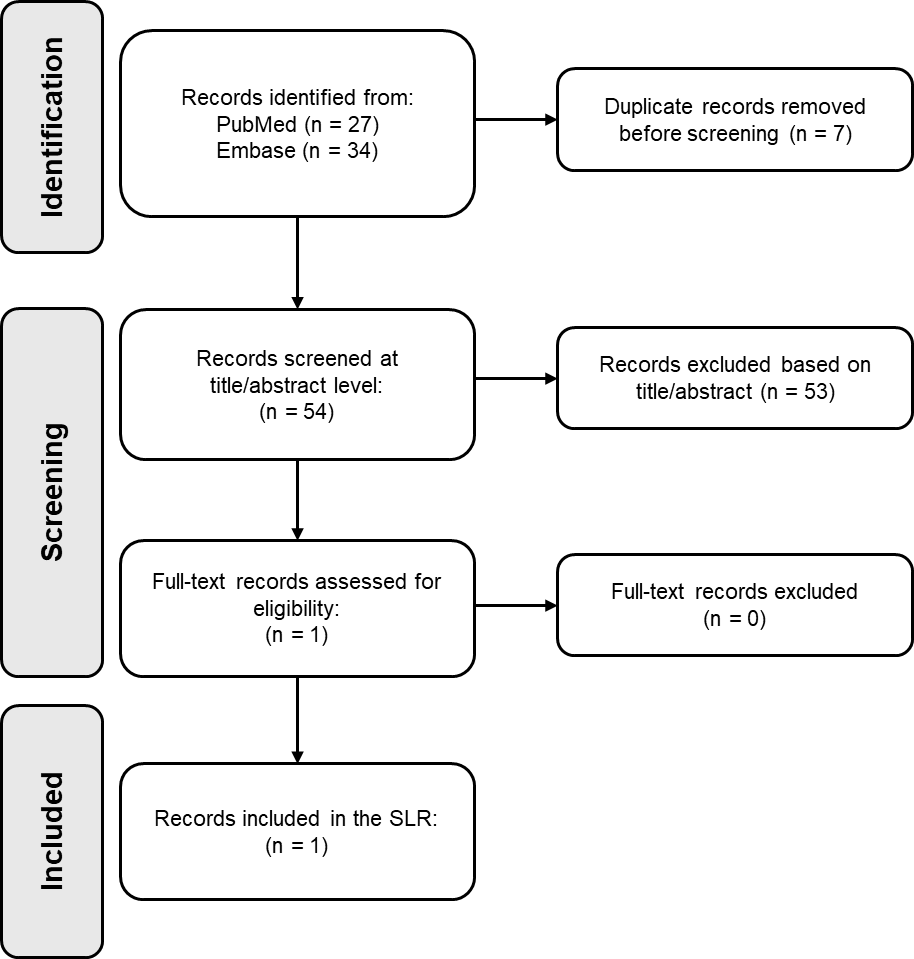


Medline (PubMed; US National Library of Medicine, National Institutes of Health) and Embase (Elsevier) search engines were used.

RCTs, Randomized clinical trials; SLR, systematic literature review; uUTI, uncomplicated urinary tract infection.
